# Gamma‐irradiated Newcastle disease virus: an alternative inactivated oncolytic virotherapy

**DOI:** 10.1111/imcb.70107

**Published:** 2026-04-05

**Authors:** Eve V Kennedy, Yimin Chuah, Amal H Mostafa, Chloe J Gates, Jade Foeng, Todd S Norton, Shaun R McColl, Iain Comerford, Justin B Davies, Farhid Hemmatzadeh, Mohammed Alsharifi

**Affiliations:** ^1^ Research Centre for Infectious Diseases, School of Biological Sciences Adelaide University Adelaide SA Australia; ^2^ Australian Nuclear Science and Technology Organisation Lucas Heights NSW Australia; ^3^ School of Animal and Veterinary Sciences Adelaide University Roseworthy SA Australia; ^4^ Present address: Todd S Norton, Precision Medicine South Australian Health and Medical Research Institute (SAHMRI) Adelaide SA Australia; ^5^ Present address: Flinders Health and Medical Research Institute Flinders University Bedford Park SA Australia

**Keywords:** Immunogenicity, inactivated virus, melanoma skin cancer, oncolytic therapy

## Abstract

Newcastle disease virus (NDV) has been investigated as an oncolytic virus in many clinical trials, demonstrating the ability of NDV to treat a range of different cancers. However, research with NDV is hindered by biosecurity risks associated with live NDV. In addition, NDV is an important poultry pathogen that is associated with widespread livestock losses and a large economic burden. While live and chemically inactivated NDV vaccines are available, they have limited efficacy and there is a need for alternative vaccines. In this study, we inactivated NDV using γ‐irradiation and tested the structural integrity, immunogenicity, and oncolytic activity of γ‐NDV using *in vitro* and *in vivo* models. Our data illustrate that the overall virion structure and protein function of γ‐NDV are well maintained. However, we did not detect neutralizing antibody responses after intramuscular or subcutaneous γ‐NDV administration in mice. While these data do not directly support the use of γ‐NDV as a vaccine candidate, our data show that γ‐NDV retained its ability to kill a range of different cancer cells *in vitro*, suggesting γ‐NDV may be a potential cancer therapeutic agent. To test this, γ‐NDV was trialed as an oncolytic therapy in a murine melanoma model. This revealed that γ‐NDV administration outperformed live NDV in terms of reduced tumor growth and overall survival. While further investigation is required to address the suitability of γ‐NDV as a poultry vaccine, our data indicate that γ‐irradiation may be a suitable inactivation method for the development of a highly effective inactivated oncolytic virotherapy.

## Graphical abstract

To overcome biological risks associated with using live NDV as an oncolytic therapy, we developed gamma‐NDV as a safer alternative. Using a murine model for skin cancer, we show that intratumor administration of gamma‐NDV outperformed live‐NDV in terms of reducing tumor growth and improving overall animal survival.

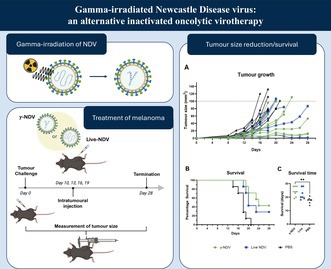

## INTRODUCTION

Newcastle disease virus (NDV) is an avian paramyxovirus that predominantly infects chickens and other domestic poultry. NDV strains are highly genetically diverse and classified according to their virulence into velogenic (highly virulent), mesogenic (medium virulence), and lentogenic (low virulence or avirulent) strains.^1^ Importantly, infections with velogenic viruses have been associated with widespread livestock losses and a large economic burden due to high mortality rates that can approach 100%.^2^, ^3^, ^4^, ^5^ Current NDV vaccines are either live‐attenuated or chemically inactivated and are mainly based on lentogenic strains. Importantly, neither vaccine type is fully effective, and vaccinated chickens can still be infected and shed viruses.^6^, ^7^ Considering the antigenic diversity and pathogenicity of NDV and the lack of effective vaccination strategies, there is an urgent need for an alternative broadly protective NDV vaccine.

We have previously reported our approach to develop highly effective γ‐irradiated vaccines such as influenza A virus (γ‐Flu)^8^, ^9^, ^10^, ^11^, ^12^, ^13^ and *Streptococcus pneumoniae (*γ‐PN).^14^, ^15^, ^16^ In addition, we demonstrated the limited impact of γ‐irradiation on the structural integrity of irradiated materials.^12^ Thus, considering the obvious need for a better NDV vaccine, we investigated the possibility of using γ‐irradiation to inactivate NDV for vaccine purposes. NDV is an ideal candidate for this approach as the large single‐stranded genome (15.2 kb) renders the virus susceptible to γ‐irradiation,^17^ and specific irradiation conditions could be optimized to ensure structural integrity and vaccine immunogenicity.^18^

Furthermore, NDV has garnered widespread attention due to its ability to selectively kill cancer cells. Several clinical trials have confirmed the ability of live NDV to reduce tumor size in a range of cancers including colorectal carcinoma,^19^ glioblastoma, breast, and ovarian cancers.^20^ However, the use of live NDV is heavily regulated as it poses a major biosecurity risk. In addition, a major shortcoming of oncolytic viruses is the induction of humoral immunity, and live NDV has been shown to induce neutralizing antibody responses in humans.^21^ Neutralizing antibodies are expected to prevent uptake into cancerous cells and consequently limit the efficacy of oncolytic viral therapy.

In this study, we illustrated the structural integrity of γ‐NDV and tested the immunogenicity in mice. Surprisingly, despite high immunogenicity, humoral responses induced by γ‐NDV are mainly nonneutralizing. Importantly, the oncolytic activity of γ‐NDV is maintained and comparable to that observed for live NDV, both in tissue culture and in animal models. Thus, the reduced ability to induce neutralizing antibody responses could be utilized to develop a new platform for solid tumor therapy based on γ‐NDV.

## MATERIALS AND METHODS

### Ethics statement

This study was conducted in compliance with the *Australian Code of Practice for the Care and Use of Animals for Scientific Purposes*. These studies were approved by the University of Adelaide Animal Ethics Committee (ethics approval numbers S‐2018‐013 and S‐2019‐108).

### Cells

Chicken embryo fibroblasts (CEFs) were prepared from 10‐day‐old chicken embryos as described previously.^22^ CEFs, Madin‐Darby canine kidney (MDCK), African Green monkey (Vero), mouse melanoma B16‐F10 (B16), and human rhabdomyosarcoma skeletal muscle cancer (RD) cells were maintained in DMEM with 10% FBS and 1% P/S. Mouse breast cancer (4T1.2) cells were maintained in RPMI with 10% FBS and 1% P/S. Human prostate cancer (PC3) cells were maintained in F12 media + 10% FBS and 1% P/S. All cells were kept at 37°C in a humidified environment with 5% CO_2_ and cells were passaged when they reached approximately 80% confluence.

### Viruses and gamma‐irradiation

Newcastle disease virus live‐attenuated V4 strain (Bioproperties, Ringwood, Victoria, Australia) was grown and titrated, as described previously.^22^ For vaccine stocks, NDV was concentrated by ultrafiltration and centrifugation at 3272× *g* using Amicon® Ultra‐15 centrifugal filter units with a 100 kDa cutoff (Merck, Bayswater, Victoria, Australia). The preparation was resuspended in phosphate‐buffered solution (PBS) and washed three times, then resuspended in PBS for the final preparation after 10‐fold concentration. NDV vaccine stocks were then aliquoted and stored at −80°C until required. Using CEFs, the titer of the vaccine stock was determined to be 2 × 10^8^ TCID_50_ mL^−1^.

Concentrated stock of NDV was irradiated at the Australian Nuclear Science and Technology Organization, NSW by exposure to 50 kGy of gamma‐irradiation from a ^60^Co source. Viruses were frozen on dry ice for the duration of irradiation and transportation. Gamma‐irradiated influenza A virus strain A/Puerto Rico/8/1937 [H1N1] (γ‐Flu) was prepared as previously described.^18^ Sterility of γ‐NDV was confirmed by three passages in eggs and Vero cells (as described and illustrated in Supplementary data 1). Structural integrity of γ‐NDV was confirmed using Haemagglutination assay, Neuraminidase assay, and electron microscopy, as previously described.^18^

### Animal studies

For vaccine immunogenicity, 6–8‐week‐old female BALB/c or C57BL/6J mice were vaccinated with 6.4 × 10^6^ TCID_50_‐equivalent/mouse for γ‐NDV or 1 × 10^6^ TCID_50_‐equivalent/mouse for γ‐Flu. For intramuscular or subcutaneous vaccination, a total volume of 50 μL per animal was used, and a volume of 32 μL was used for intranasal vaccination. Mice were first anesthetized by intraperitoneal injection of 10% ketamine and 1% xylazine prior to vaccination. Prime‐boost vaccination strategy included vaccine administration at 2‐ or 3‐week intervals, and some experiments involved co‐administration of γ‐NDV with γ‐Flu, aluminium hydroxide (alum, 10%), Poly(I:C) (50 μg per mouse), or incomplete Freund's adjuvant (IFA, 10%). Blood samples were collected at specific time points postvaccination by either submandibular bleeding or cardiac puncture after mice were humanely euthanized. Enzyme‐linked immunosorbent assay (ELISA) was used to measure IgG responses in serum samples, as described previously.^18^ For NDV‐specific antibody responses, ELISA plates were coated with NDV antigen. In addition, we used Focus‐Forming Inhibition Assays (FFIA) to measure the ability of immune sera to neutralize NDV, as described previously.^12^

For tumor therapy studies, 6–8‐week‐old female C57BL/6J mice were anesthetized with isofluorane (2% isofluorane with 2 L min^−1^ O_2_) and 150 μL of B16 murine melanoma cells (1 × 10^5^ cells per mouse) in PBS were subcutaneously injected. Mice were monitored daily for the development of tumors and clinical disease symptoms, and tumor size was measured with calipers every second day from the time when tumors became palpable. Mice were humanely euthanized when tumors reached 100 mm^2^ or at day 28 posttumor induction if mice had not reached the tumor size cutoff. For NDV therapy, 10^7^ TCID_50_ per mouse of live NDV or equivalent for γ‐NDV diluted in PBS was intratumorally injected in a total volume of 50 μL, and neat PBS was intratumorally injected as a control.

### MTT assay

Different cancer cell lines were plated in 96‐well flat‐bottom plates at 5 × 10^4^ cells per well and allowed to adhere overnight using similar conditions as described above. Live or γ‐NDV was activated with 10 μg mL^−1^ of Tosyl phenylalanyl chloromethyl ketone (TPCK)‐trypsin (Sigma‐Aldrich, Bayswater, Victoria, Australia) at 37°C for 30 min and added to the cells at a multiplicity of infection (MOI) of 20. Cells were incubated for 24 or 48 h, then washed three times with PBS, and fresh media was added. Ten microliters of 5 mg mL^−1^ MTT (3‐(4,5‐Dimethylthiazol‐2‐yl)‐2,5‐Diphenyltetrazolium Bromide) (Thermofisher, Scoresby, Victoria, Australia) was added to each well and plates were incubated for 4 h at 37°C. Media was removed and 50 μL dimethyl sulfoxide (Sigma‐Aldrich, Bayswater, Victoria, Australia) was added to each well to dissolve the formazan crystals. To measure cell viability, the absorbance was measured at 562 nm using a Bio‐Tek Instruments plate reader and percentage cytotoxicity was calculated using the formula [1−(absorbance treated cells/absorbance untreated cells) × 100].

### Giemsa staining

B16 cells and 4T1.2 cells were treated with live or γ‐NDV as described for MTT assay, but using an MOI of 10. Cells were incubated for 24 h prior to washing with PBS and fixed with methanol for 10 min at room temperature. Methanol was removed, and plates were allowed to air dry. Cells were then stained with Giemsa (Sigma 32884, 1:20 dilution in MilliQ water). Cells were washed with PBS and visualized with an Olympus phase‐contrast ULWCD 0.30 microscope.

### GFP‐Split assay

To confirm syncytia formation, we used two plasmids synthesized by Vector Builder expressing two portions of the GFP protein: pCMV(nc‐GFP1‐7‐SpyTag):IRES:EBFP and pCMV>(cc‐GFP8‐11‐SpyCatcher2):IRES:CyPet. B16 or 4T1.2 cells were plated as described above, and transfected using 100 ng of either plasmid using lipofectamine 2000 (ThermoFisher, Scoresby, Victoria, Australia) in Opti‐MEM™ I Reduced Serum Medium (ThermoFisher, Scoresby, Victoria, Australia) for 6 h at 37°C in 5% CO_2_. Transfected cells were then washed with PBS, trypsinized and mixed in a 1:1 ratio. Live and γ‐NDV were activated with 10 μg mL^−1^ of TPCK‐trypsin and added to transfected cells at an MOI of 5 and incubated for 24 h at 37°C in 5% CO_2_. For visualization, cells were fixed with 1% cold paraformaldehyde for 20 min at room temperature in the dark and then stained with DAPI. Plates were visualized using Nikon TiE inverted fluorescence microscope and analyzed using NIS elements software (Tokyo, Japan).

### Statistical analyses

Quantitative results were expressed as mean ± s.e.m. Unpaired Student's *t*‐test was used for comparison of data from two separate groups, and one‐way ANOVA was used for comparison of data from three or more groups involving a single independent variable. All analyses were performed using GraphPad Prism, version 10 (GraphPad Software, La Jolla, CA, USA). *P*‐values <0.05 (95% confidence interval) were considered statistically significant.

## RESULTS

### Sterility and structural integrity of γ‐NDV

**Figure 1:**
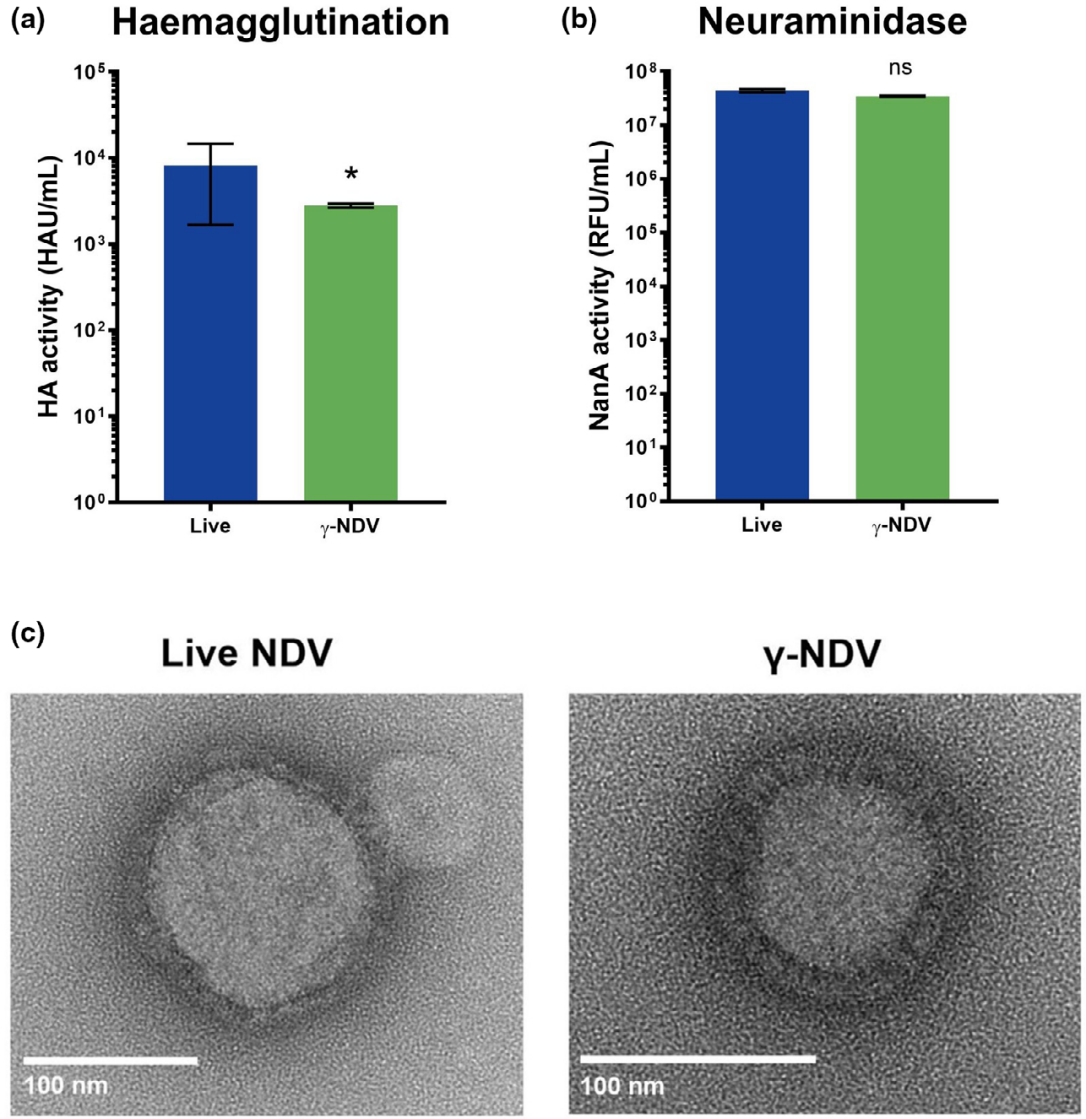
Structure of Newcastle disease virus (NDV) is maintained after γ‐irradiation. NDV was inactivated by exposure to 50 kGy of γ‐irradiation and the **(a)** haemagglutinin activity, **(b)** neuraminidase activity, and **(c)** whole virion structure of live and γ‐irradiated NDV were analyzed. Haemagglutinin activity was measured by the ability of the virus to agglutinate RBCs. Neuraminidase activity was measured by cleavage of 4‐MUNANA into the fluorescent substrate 4‐MU and the relative fluorescent intensity was measured for each sample. Live NDV was used as a positive control. For whole virion structure, live and γ‐NDV were imaged using a transmission electron microscope. Quantitative data are expressed as mean ± s.e.m. (*n* = 6, collated from two independent experiments). Data were analyzed by one‐way ANOVA (*P < 0.05, ns = not significant).

In this study, we investigated the oncolytic activity of γ‐NDV. Sterility of γ‐NDV was confirmed by lack of infectivity in embryonated eggs and Vero cell culture (Supplementary figure 1). To assess the structural integrity of γ‐NDV, we initially examined the functional activity of the surface protein haemagglutinin‐neuraminidase (HN). As shown in Figure 1a, γ‐NDV retained high hemagglutination titer illustrating the ability of HN protein to bind and cross‐link sialic acid receptors on red blood cells (RBCs). Additionally, we tested the neuraminidase activity by measuring cleavage of 4‐MUNANA into the fluorescent substrate 4‐Methylumbelliferyl (4‐MU). Our data illustrated that the neuraminidase activity of HN was not affected by γ‐irradiation as comparable levels of enzymatic activity were observed for both live and γ‐NDV (Figure 1b). Finally, we used electron microscopy to illustrate that γ‐NDV is structurally intact (Figure 1c).

### Limited neutralizing antibody responses despite high immunogenicity

**Figure 2:**
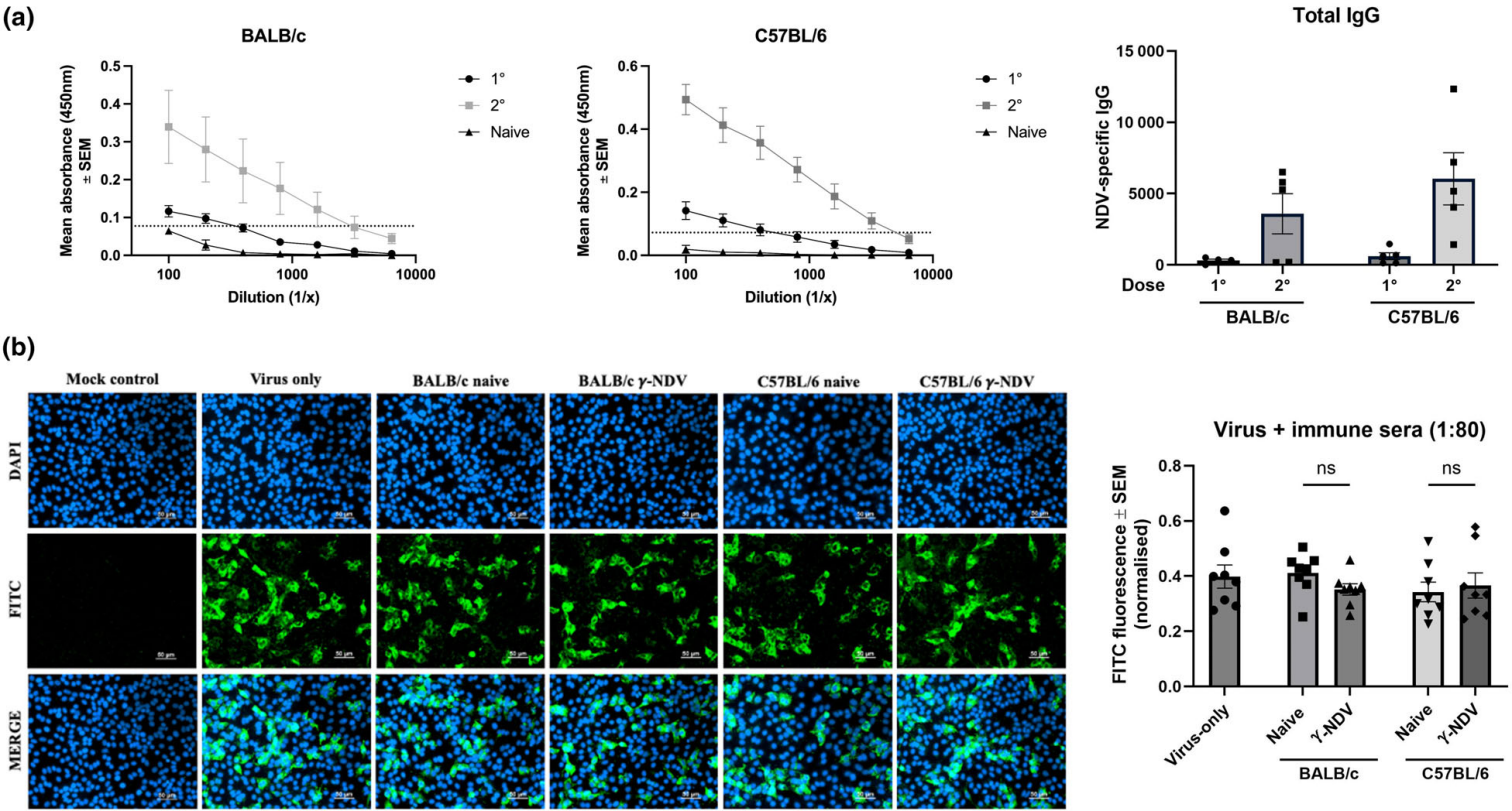
Lack of neutralizing antibody responses following vaccination with γ‐Newcastle disease virus (NDV). C57BL/6J and BALB/c mice were vaccinated subcutaneously with two doses of 10^7^ TCID_50_ per mouse γ‐NDV 3 weeks apart. **(a)** Serum samples were collected 13 days after each vaccination dose, tested for NDV‐specific total IgG, and titres were calculated based on a cutoff (dotted line) determined from the mean + 3 × sd. of the naive control absorbance values at a 1/100 dilution. Data are presented as mean ± s.e.m. **(b)** Serum samples collected 3 weeks after the secondary dose were tested for neutralizing antibody responses using FFIA and representative images are shown for a 1:80 serum dilution. Quantitative data are presented as mean ± s.e.m. (*n* = 8 using technical replicates, collated from two independent experiments) and analyzed by one‐way ANOVA (ns = not significant).

Initially, to assess the immunogenicity of γ‐NDV, BALB/c mice were vaccinated twice intramuscularly with γ‐NDV at 3‐week intervals, and serum samples were collected 3 weeks after each vaccination. Presence of NDV‐specific IgG was determined by direct ELISA, with titres being calculated relative to naïve serum (Supplementary figure 2A). We performed FFIA to measure neutralizing antibody responses (nAbs) and, surprisingly, our data indicated that the anti‐NDV IgG responses obtained after prime‐boost vaccination did not correlate with an increase in the ability of the immune sera to neutralize NDV infectivity. This was shown by the lack of significant reduction in the intensity of fluorescence following treatment of NDV with immune sera prior to infecting monolayers of Vero cells compared to NDV treated with naïve serum (Supplementary figure 2B, C). To confirm that these results were not dependent on the route of vaccination or inbred mouse strain used, we tested the immunogenicity of γ‐NDV following subcutaneous administration into BALB/c and C57BL/6J mice. Mice were treated twice with γ‐NDV at 2 weeks intervals and immune sera were harvested 2 weeks after each treatment and tested for IgG responses and NDV neutralization. Our data clearly show that despite the induction of strong antibody responses following vaccination with γ‐NDV (Figure 2a), no nAbs against NDV were observed in either BALB/c or C57BL/6J mice (Figure 2b). Therefore, we investigated the possible use of adjuvants to induce NDV‐specific nAbs.

**Figure 3:**
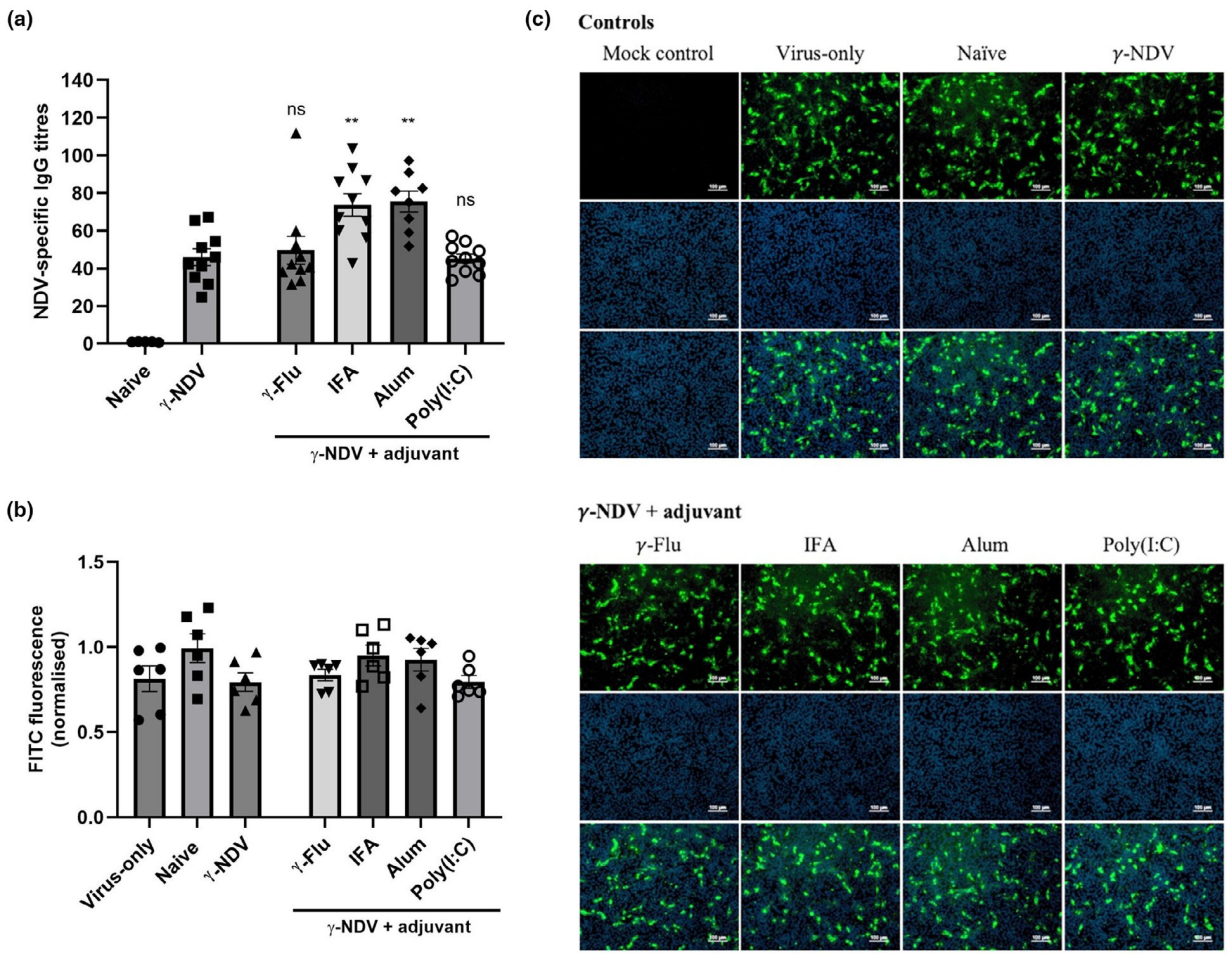
The effect of adjuvants on antibody responses to γ‐Newcastle disease virus (NDV). BALB/c mice were vaccinated twice intramuscularly with γ‐NDV ± adjuvants. Two weeks after the second immunization, serum was harvested. **(a)** Serum samples were tested for NDV‐specific IgG responses using ELISA and presented as fold change relative to naïve serum (*n* = 10 biological replicates, collated from 2 independent experiments). **(b)** Neutralizing antibody responses were determined by FFIA and a fluorescence microscope was used to quantify FITC‐fluorescence (representative of NDV infection) relative to DAPI‐fluorescence (cell nuclei). **(c)** Representative images are shown at a 1:10 serum dilution (representative of 3 wells per group). Data are presented as mean ± s.e.m. (*n* = 6 using technical replicates, collated from two independent experiments) and analyzed by one‐way ANOVA. Adjuvanted IgG responses were compared to IgG responses generated by γ‐NDV alone (**P < 0.01, ns = not significant).

We have previously demonstrated the adjuvant activity of γ‐Flu when co‐administered with other γ‐irradiated vaccines.^15^, ^23^ Therefore, we investigated the impact of γ‐Flu+γ‐NDV co‐administration on NDV‐specific responses. In order to circumvent competition for receptor binding between γ‐Flu and γ‐NDV, other commonly used adjuvants were also tested alongside. Mice were vaccinated twice 2 weeks apart with γ‐NDV alone or combined with either Incomplete Freund's adjuvant (IFA), alum, poly(I:C), or γ‐Flu as an adjuvant. Serum samples were collected 2 weeks after the second vaccination and tested for NDV‐specific antibody responses. As shown in Figure 3a, IgG responses were enhanced when γ‐NDV was co‐administered with IFA and alum. In contrast, neither poly(I:C) nor γ‐Flu enhanced the immunogenicity of γ‐NDV. Interestingly, despite the enhanced IgG responses following vaccination with γ‐NDV + IFA or alum, we did not detect a significant increase in NDV‐specific nAbs (Figure 3b, c). There was no significant difference in normalized FITC‐fluorescence (indicative of NDV replication) when comparing serum from mice treated with γ‐NDV alone to any of the adjuvanted γ‐NDV groups (Figure 3b). Collectively, these data indicate that γ‐NDV does not induce nAbs, despite high immunogenicity.

### Oncolytic activity of γ‐NDV

**Figure 4:**
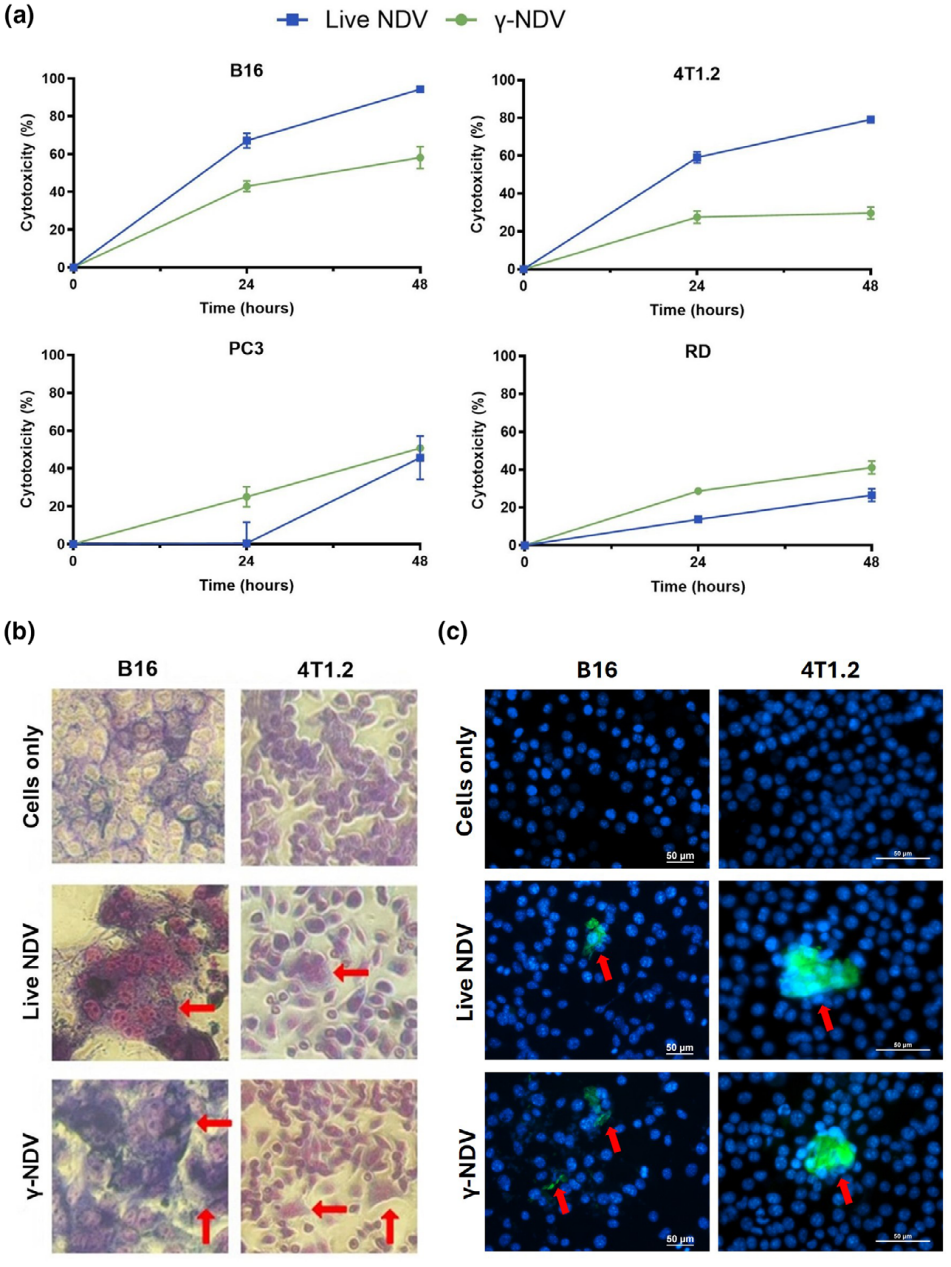
Cytotoxicity of live and γ‐Newcastle disease virus (NDV). **(a)** MTT assay was used to measure cell death in mouse melanoma B16 cells, mouse breast cancer 4T1.2 cells, human prostate cancer PC3 cells, and human muscle cancer RD cells. Cell monolayers were treated with live or γ‐NDV at MOI of 20, and absorbance was measured at 562 nm. Cytotoxicity was measured using the formula [1−(absorbance treated cells/absorbance untreated cells) × 100] and presented as mean ± s.e.m. (*n* = 3). **(b)** Syncytia formation was observed using Giemsa stain to visualize multi‐nucleated cells in both B16 and 4T1.2 cell monolayers after treatment with live or γ‐NDV, compared to untreated controls. **(c)** Cell fusion was illustrated using a GFP‐split assay where different batches of transfected cells were mixed and treated with live or γ‐NDV. Cells were incubated and analyzed for GFP signal using a fluorescence microscope.

The reduced level of nAbs induced by γ‐NDV highlighted the possible use of γ‐NDV as an effective oncolytic therapy. To test this possibility, we used MTT assays to measure cytotoxicity in mouse melanoma (B16), mouse breast cancer (4T1.2), human prostate cancer (PC3), and human muscle cancer (RD) cells induced by live and γ‐NDV at 24 and 48 h posttreatment (Figure 4a). We also investigated the cytotoxic effect of γ‐NDV against a variety of other murine and human cancer cells, with cytotoxicity measured at 48 h posttreatment (Supplementary table 1). Collectively, our data illustrate the effectiveness of γ‐NDV in terms of inducing cytotoxicity against a range of different cancers, including solid and blood cancers, despite lacking the ability to replicate.

Syncytia formation is a possible mechanism for oncolysis and based on the structural integrity of γ‐NDV it is expected that γ‐NDV would be able to fuse with host cells and induce syncytia formation among adjacent cells. Monolayers of B16 and 4T1.2 cells were treated with live or γ‐NDV for 24 h and fixed and stained with Giemsa to visualize multinucleated cells (Figure 4b). In addition, to confirm the ability of γ‐NDV to induce cellular fusion, we used a GFP‐split system as shown in Supplementary figure 3. Upon fusion of two cells which each carry a different plasmid, the full GFP protein will be assembled, and a signal will be detected. Clear GFP signals were detected when transfected cancer cell populations were treated with either live or γ‐NDV preparations (Figure 4c), indicating the ability of γ‐NDV to induce syncytia formation.

**Figure 5:**
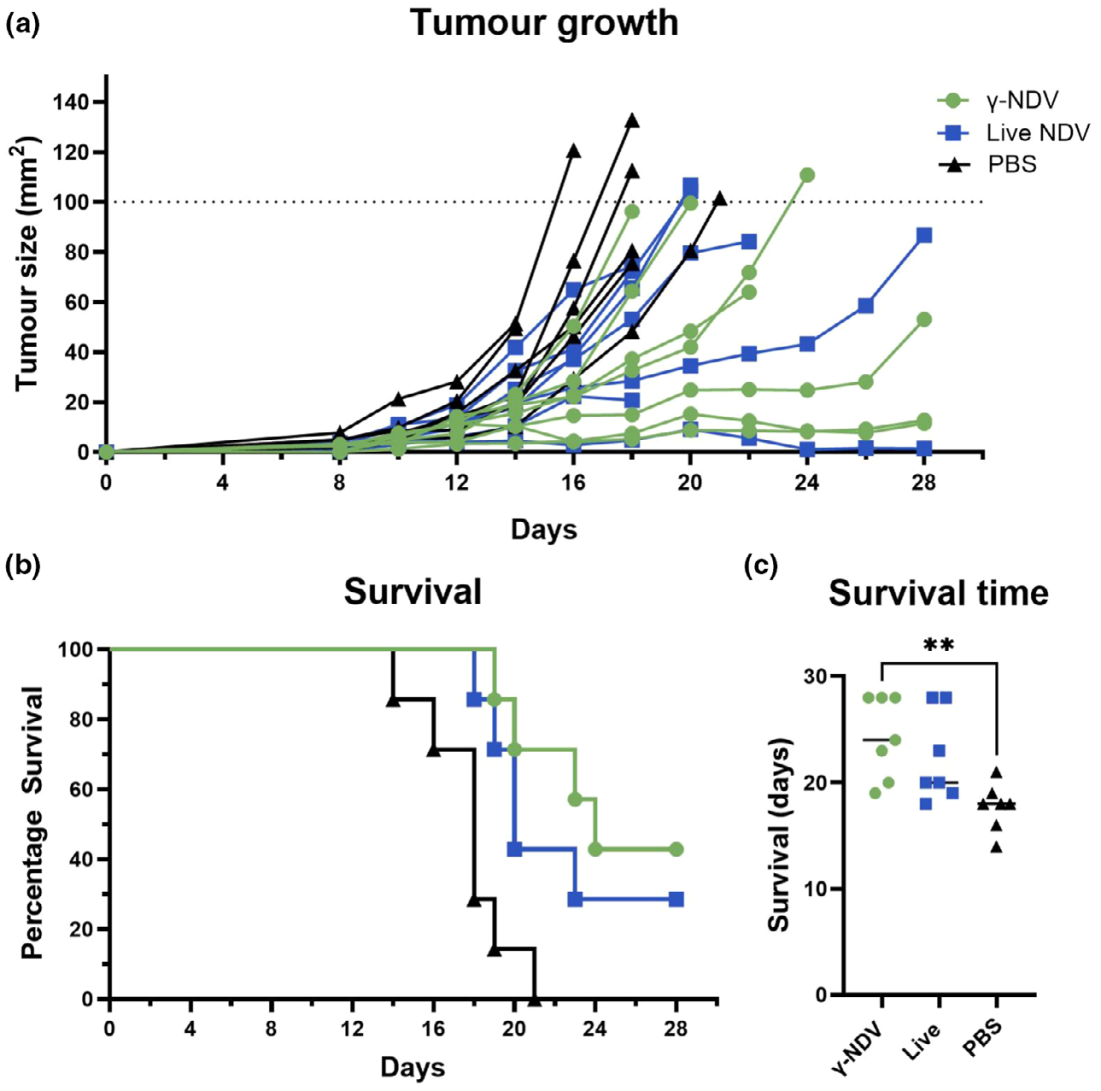
Treatment of B16 tumors with γ‐Newcastle disease virus (NDV). B16.F10 cells were injected subcutaneously into C57BL/6J mice. Tumors were treated with live NDV, γ‐NDV, or PBS on days 10, 13, 16, and 19. **(a)** Tumors were measured every second day with calipers and mice were humanely euthanized when tumor size reached 100 mm^2^ (dotted line) or at day 28 posttumor injection as the experimental endpoint. **(b)** Percentage survival and **(c)** survival time in days were measured. Data are presented as mean ± s.e.m. (*n* = 7 per group). For **(a)** and **(c)**, data were analyzed by one‐way ANOVA comparing all groups (**P < 0.01).

To address the oncolytic activity of γ‐NDV *in vivo*, B16 cells were injected subcutaneously into C57BL/6J mice, and tumor‐bearing mice were treated intratumorally with live or γ‐NDV on days 10, 13, 16, and 19 posttumor induction. Injection of PBS was used as a negative control. Tumor size was measured every second day to monitor the impact of different therapies (Figure 5a). At d16 and d18 posttumor injection, there was a significant reduction in tumor sizes observed for mice treated with γ‐NDV compared to mice treated with live NDV, indicating that treatment with γ‐NDV was more effective than treatment with live NDV. Mice were humanely euthanized when tumor size reached 100 mm^2^ or by the experimental endpoint (d28‐posttumor injection). Notably, a higher percentage of mice treated with γ‐NDV (43%) and live NDV (29%) survived compared to control untreated mice, where no mice survived past day 21 posttumor injection (Figure 5b). Furthermore, when overall survival time was considered, a significantly prolonged survival time was observed in the γ‐NDV‐treated group compared to untreated PBS‐control mice (Figure 5c). The mean survival time for untreated mice was 18 days compared to 20 and 24 days for mice treated with live and γ‐NDV, respectively. No significant differences in survival rate were observed when comparing live NDV and γ‐NDV treatment, suggesting that γ‐NDV may be a viable alternative to enhance safety and efficacy of virotherapy.

### Impact of intratumoral administration on NDV‐specific antibody responses

**Figure 6:**
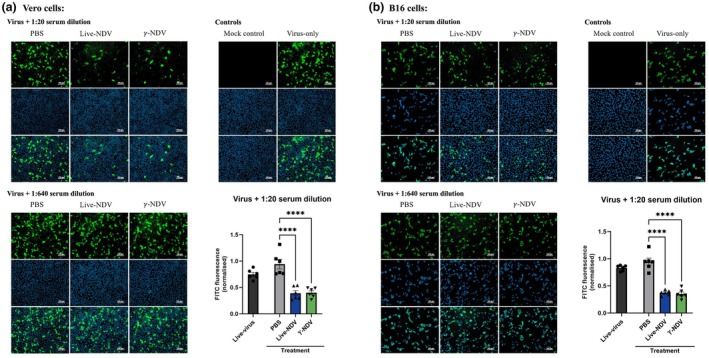
Neutralizing antibody responses in tumor‐bearing mice. B16.F10 tumors were induced by subcutaneous injection of B16.F10 cells into C57BL/6J mice. Mice were then treated intratumorally with live Newcastle disease virus (NDV), γ‐NDV, or PBS at days 10, 13, and 16 posttumor injection, and serum was collected at day 20 posttumor injection. To measure neutralization, FFIA was performed using **(a)** Vero cells and **(b)** B16 cells. Plates were stained with DAPI (cell nucleus) and FITC (NDV infection), then visualized using a fluorescence microscope. FITC relative to DAPI was quantified as a measure of neutralization. Representative images are shown at a 1:20 and 1:640 serum dilution (representative of three wells tested per group). Quantitative data at the 1:20 serum dilution are presented as mean ± s.e.m. (*n* = 6 using technical replicates, collated from two independent experiments) and analyzed by one‐way ANOVA (****P < 0.0001).

To address the impact of intratumoral injection on the immunogenicity of γ‐NDV, B16‐tumor bearing mice were treated with live NDV, γ‐NDV, or PBS at days 10, 13, and 16 posttumor injection and serum samples were harvested at day 20 posttumor injection and tested for nAbs. Live NDV was treated with pooled immune sera from tumor‐bearing mice that had been treated with live NDV, γ‐NDV, or PBS and FFIAs were performed using Vero and B16 cells (Figure 6). Surprisingly, we found that serum samples collected from tumor‐bearing C57BL/6J mice treated with γ‐NDV were able to neutralize NDV as measured by reduced NDV infectivity in both Vero and B16 cells. This is contradictory to our earlier data that showed limited nAbs in both BALB/c mice and C57BL/6J mice (Figure 2). It is important to note that in the B16 tumor model, C57BL/6J mice received 10^7^ TCID_50_‐equivalent per mouse at three time points administered 3 days apart, in contrast to using two injections of a similar dose at 2 weeks intervals for immunogenicity testing. Therefore, increased antigen load or potentially specific signals in the tumor microenvironment may have affected the immune response to γ‐NDV leading to the induction of nAbs in these settings.

## DISCUSSION

NDV is an important avian pathogen that is associated with large livestock losses and a great economic burden globally. Considering the limited efficacy of current live attenuated and chemically inactivated vaccines against highly pathogenic NDV strains, we investigated the immunogenicity of γ‐irradiated NDV. Previous studies demonstrated γ‐irradiation to be a superior inactivation method compared to chemical inactivation in terms of maintaining structural integrity and induction of effective immune responses.^10^, ^24^, ^25^ Our data illustrated that γ‐NDV had high levels of structural integrity as evidenced by HA activity similar to nonirradiated NDV and generated high IgG responses. However, the strong IgG responses observed after γ‐NDV administration did not translate to virus neutralization. Therefore, we addressed the possibility of using adjuvants to enhance the immunogenicity of γ‐NDV. Poly(I:C) and γ‐Flu provide adjuvant activity in a similar manner through stimulation of IFN‐I responses,^11^, ^26^ whereas alum and oil adjuvants act as stabilizers and can help recruit immune cells to the immunization site.^27^, ^28^ Furthermore, alum shifts the immune responses to a T_H_2 response that is characterized by enhanced antibody responses.^28^, ^29^, ^30^ However, despite the use of different adjuvants, no nAbs were observed following administration of γ‐NDV. It is important to note that infection of chickens and mice with NDV induces both antibody and T‐cell responses.^31^, ^32^, ^33^, ^34^ Thus, further investigations should address the ability of γ‐NDV to induce T‐cell responses. Nonetheless, considering the important role of nAbs, our data indicated that γ‐NDV may not be a suitable vaccine candidate for poultry. However, the structural integrity of γ‐NDV opens an avenue for its use as an oncolytic virotherapy.

The use of oncolytic viruses to target cancers is a promising area of research, but it is associated with biosecurity risks related to using pathogenic viruses. Consequently, γ‐NDV represents a unique approach where inactivated virus can be used as a virotherapy without an outbreak risk. In mammalian cells, NDV is a potent activator of innate signaling, which can lead to the activation of both intrinsic and extrinsic apoptotic pathways. Pro‐apoptotic signaling molecules induced in cancer cells by live NDV include IFNα,^35^ TRAIL,^36^ TNFα,^37^ and IFNβ.^38^ Moreover, HN, fusion (F), and matrix (M) proteins are all present in γ‐NDV and our data demonstrate that both HN and F are functional. It has been reported that loss of sialic acids on the cancer cell surface can impair oncolytic activity, highlighting the role of HN binding to sialic acid.^39^ Furthermore, studies have also shown that NDV surface antigens HN and F lead to cell death when expressed on the surface of tumor cells or CEF DF1 cells.^40^, ^41^, ^42^, ^43^ This is abrogated by HN‐specific nAbs, but not F‐specific nAbs.^41^ Furthermore, M protein alone has been shown to be oncolytic as it harbors a BH3 domain which shares homology with Bcl2 proteins, thus enabling the M protein to interact with Bax and activate the intrinsic pathway of apoptosis.^44^ Overall, the ability of different viral proteins to induce oncolysis suggests a multifaceted mechanism of oncolysis. In fact, our GFP‐split assay clearly confirms the ability of γ‐NDV to induce syncytia formation in cancer cells. It is important to note that sialic acid receptors are cellular entry receptors for NDV, and that sialic acids are upregulated in some cancers (reviewed in^45^). These features may allow NDV and γ‐NDV to specifically target cancer cells. Furthermore, enhanced sialic acids on cancer cells has been identified as a cause of chemotherapy resistance, specifically against cis‐platin.^46^ Thus, NDV could specifically target resistant cancer cells, and the susceptibility of chemotherapy resistant cells to NDV could be utilized to develop combination therapies wherein γ‐NDV is co‐administered with cis‐platin.

Interestingly, immune sera from tumor‐treated animals show nAbs following intratumoral administration of γ‐NDV, in contrast to intramuscular and subcutaneous administration into healthy animals. The difference in nAbs may be caused by a higher antigen dose used for tumor treatment, inflammatory responses, and possible modification of viral proteins within the tumor microenvironment. Thus, further investigations should address the underlying mechanism for the induction of nAbs in this model, which may facilitate the development of γ‐NDV as a poultry vaccine. Importantly, this study demonstrates that NDV does not need to replicate to induce an oncolytic effect, with γ‐NDV outperforming live NDV in terms of antitumor efficacy in a B16 murine melanoma model. While this proof‐of‐concept was based on the lentogenic V4 strain, highly virulent strains that are expected to elicit stronger immune responses can be utilized as γ‐irradiated oncolytic therapy to overcome biosecurity regulations related to live NDV.

## AUTHOR CONTRIBUTIONS

MA and EVK conceived and designed the study. EVK, YC, AHM, CJG, and JF performed experiments and prepared reagents. EVK, YC, and AHM performed statistical analyses. MA, EVK, YC, AHM, JF, TSN, SRM, IC, JBD, and FH wrote the manuscript.

## CONFLICT OF INTEREST

The authors have no conflicting financial interests.

## Supporting information


**Supplementary** **data** **1**

## Data Availability

The data that support the findings of this study are available from the corresponding author upon reasonable request.
