# Supplementary material for: Gamma‐irradiated Newcastle disease virus: an alternative inactivated oncolytic virotherapy
Source: Immunol Cell Biol. 2026 Apr 5;104(5):473–84. doi: 10.1111/imcb.70107 (PMC13155051; doi:10.1111/imcb.70107)
Supplement: Supplementary file 1 — Supplementary data 1 [file IMCB-104-473-s001.docx]

# **Gamma-irradiated Newcastle Disease virus: an alternative inactivated oncolytic virotherapy**

**Authors**

Eve V. Kennedy^1^, Yimin Chuah^1^, Amal H. Mostafa^1^, Chloe J. Gates^1^, Jade Foeng^1^, Todd S. Norton^1^, Shaun R. McColl^1^, Iain Comerford^1^, Justin B. Davies^2^, Farhid Hemmatzadeh^3^, and Mohammed Alsharifi^1*^

**Affiliations**

^1^ Research Centre for Infectious Diseases, School of Biological Sciences, Adelaide University, Adelaide, SA 5005, Australia.

^2^ Australian Nuclear Science and Technology Organisation, Lucas Heights, NSW 2234, Australia.

^3^ School of Animal and Veterinary Sciences, Adelaide University, Roseworthy, SA 5371, Australia.

***Corresponding author**

A/Prof. Mohammed Alsharifi,

Research Centre for Infectious Diseases, School of Biological Sciences, Adelaide University, Adelaide, SA 5005, Australia.

[mohammed.alsharifi@adelaide.edu.au](mailto: mohammed.alsharifi@adelaide.edu.au).

**Supplementary materials**

**A: Sterility testing of γ-NDV: in embryonated chicken eggs**

|  | **Passage 1** | **Passage 2** | **Passage 3** |
| --- | --- | --- | --- |
| **Live NDV** | +++++ | +++++ | +++++ |
| **γ-NDV** | ----- | ----- | ----- |
| **PBS** | ----- | ----- | ----- |

**B: Sterility testing of γ-NDV in Vero cells.**

**Supplementary figure 1. Sterility testing of γ-NDV.**

Sterility of irradiated preparations was confirmed by three passages in eggs and Vero cells.

1. 10-day-old embryonated chicken eggs were inoculated with live NDV, γ-NDV or PBS and were then incubated for 48 hours at 37°C. Allantoic fluid was then harvested and used to infect fresh 10-day-old eggs (Passage 2). This was repeated for a 3^rd^ Passage. Harvested allantoic fluid was then tested for NDV infection by haemagglutination assay (“+” indicates allantoic fluid could haemagglutinate RBCs and “-” indicates no haemagglutination). 5 eggs were infected per group per passage.
2. Live and γ-NDV were activated with 1μg/mL trypsin at 37°C for 1 hour. Monolayers of Vero cells were then treated with the preparations at MOI of 40 and incubated for 24 hours. As a control, monolayers were also treated with allantoic fluid from uninfected 12-days old embryonated eggs. Supernatant was collected and used to infect fresh Vero cell monolayers and incubated for a further 24 hours. This was then repeated for a third passage, and cells were then fixed and stained with DAPI to visualise cell nuclei and treated with chicken anti-NDV and then washed and stained with FITC-conjugated anti-chicken IgY antibodies to visualise NDV-infected cells. Infection levels of Passage 3 are shown here. Images are representative of 3 wells per sample, and 2 independent experiments.

**
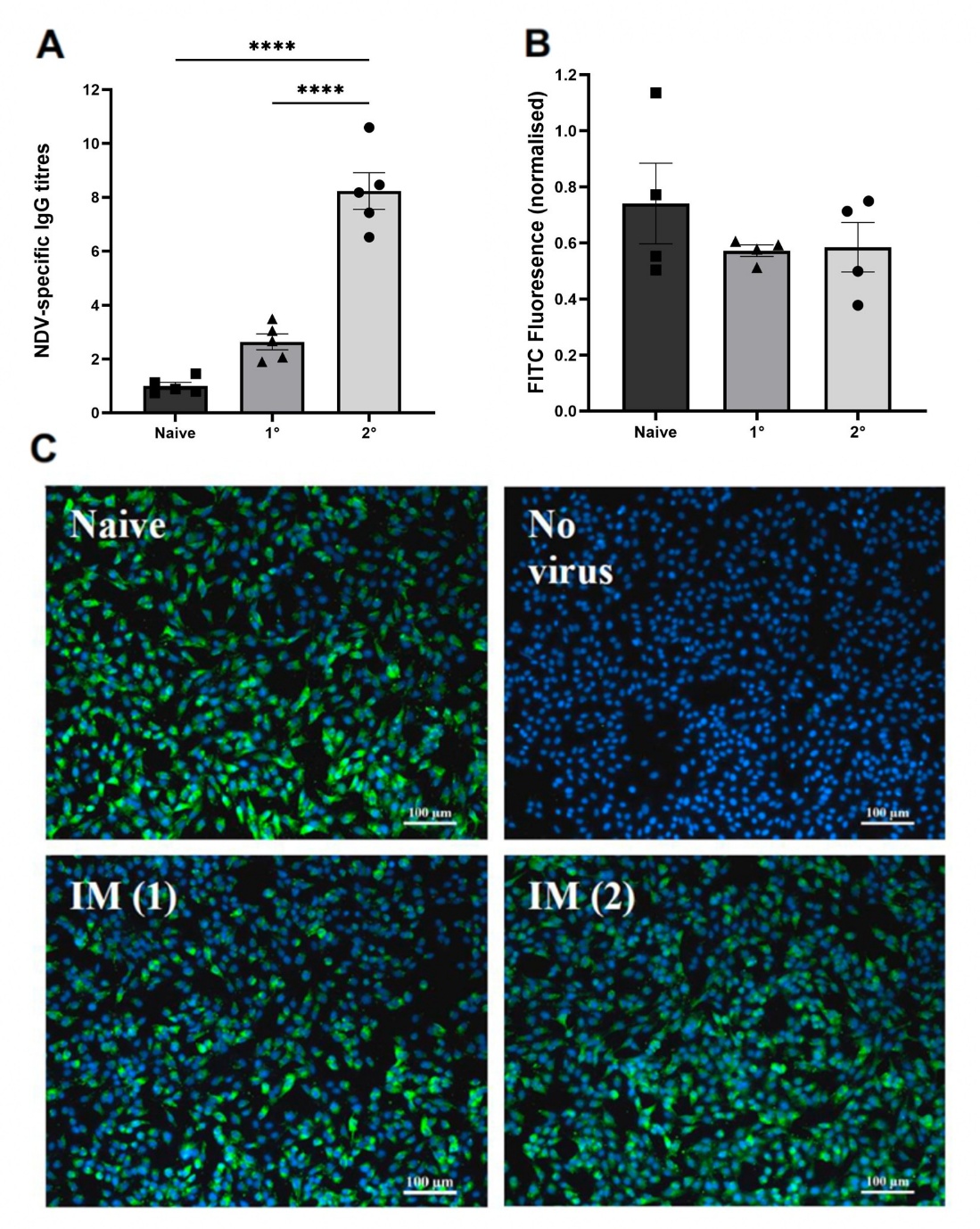
**

**Supplementary figure 2. Lack of neutralising antibody responses following intramuscular injection of γ-NDV.**

BALB/c mice were immunised intramuscularly with two doses of γ-NDV (10^7^ equivalent TCID_50_/dose) three weeks apart and serum samples were collected 20 days after each vaccination with naïve unvaccinated animals used as controls. (A) NDV-specific IgG responses were determined by direct ELISA using NDV as a coating antigen and expressed as a fold change relative to naïve serum. (B) Neutralising antibody responses were determined by FFIA and a fluorescence microscope was used to quantify FITC-fluorescence (representative of NDV infection) relative to DAPI-fluorescence (cell nuclei). (C) Representative images were also taken of each sample at a 1:10 serum dilution (representative of 3 wells tested per serum group). Quantitative data are presented as mean ± SEM and analysed by one-way ANOVA (**** p < 0.0001).

**Table 1. Cytotoxicity of γ-NDV in different cancer cell lines.** Cells were treated with γ-NDV or untreated (control). MTT assay was performed 48 hours later and used to calculate cytotoxicity. Human lung cancer (A549) cells were maintained in DMEM with 10% FBS and 1% P/S. Human melanoma (C32), human leukaemia (HL-60), human breast cancer (MDA), mouse T cell lymphoma (EL-4), mouse B cell leukaemia (L1210) and mouse mastocytoma (P815) cells were maintained in RPMI with 10% FBS and 1% P/S.

| **Cell line** | **Description** | **Cytotoxicity at 48 hours (%)** |
| --- | --- | --- |
| C32 | Human melanoma | 28.9 ± 2.00 |
| MDA-MB-231 | Human breast cancer | 44.0 ± 2.51 |
| A549 | Human lung cancer | 22.1 ± 7.43 |
| HL-60 | Human leukaemia | 41.8 ± 6.49 |
| EL-4 | Mouse T cell lymphoma | 65.8 ± 0.45 |
| L1210 | Mouse B cell leukaemia | 33.1 ± 3.02 |
| P815 | Mouse mastocytoma | 9.43 ± 3.56 |

**
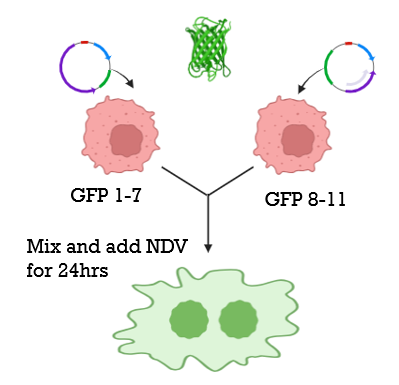
**

**Supplementary figure 3. A schematic of the GFP-split system for syncytia visualisation.** 4T1.2 or B16 cancer cells were divided into 2 batches and each batch was transiently transfected with plasmids encoding different halves of the GFP protein (either GFP 1-7 or GFP 8-11). These cells were then mixed and treated with either live or γ-NDV at MOI 5 for 24hrs. Upon fusion between cells carrying the different plasmids, the GFP molecule will be complete and a GFP signal can be picked up to confirm the fusion.

**Supplementary method section:**

**Haemagglutination assay (HA)**

Irradiated and non-irradiated NDV were serially diluted in normal saline (0.85%) in 96-well round-bottomed microtitre plates. RBCs (0.6%) were added to each well and plates were scored for mesh or pellet formation. The reciprocal of the highest virus dilution to give a positive reading was considered the haemagglutination units (HAU).

**Neuraminidase assay (NA)**

Irradiated and non-irradiated NDV preparations were serially diluted in PBS and 25μL of each dilution was added to microtitre plates. 25μL of 0.125mM 2’-(4-Methylumbelliferyl)-α-D-N-acetylneuraminic acid (4-MUNANA, Sigma M8639) was added to virus samples. Active neuraminidase cleaves 4-MUNANA into the fluorescent substrate 4-Methylumbelliferyl (4-MU). Plates were incubated in the dark at 37°C for 1 hour with gentle shaking every 15 minutes to facilitate cleavage of 4-MUNANA. Reaction was stopped with ice-cold 0.5M Na_2_CO_3_ (pH 10.5) and relative fluorescence was measured using a SpectraMax fluorescent plate reader with an excitation wavelength of 365nm and an emission wavelength of 450nm.

**Enzyme-Linked Immunosorbent Assay (ELISA)**

Serum samples were assayed by ELISA to determine NDV-specific IgG responses. 96-well high-binding ELISA plates (Corning) were coated with live-NDV diluted in Na_2_CO_3_/NaHCO_3_, and incubated overnight at room temperature. The plates were then washed 3 x with washing buffer (PBS + 0.05% Tween), blocked with skin milk (2% w/v), and incubated for 2 hours at room temperature. Immune sera were serially diluted in PBS, and added to the washed plates, followed by another incubation at room temperature for 2 hours. Subsequently, horseradish peroxidase (HRP)-conjugated goat anti-mouse total IgG (Invitrogen) diluted in blocking buffer was added, followed by colour-developing solution (1:1 ratio of H_2_O_2_ and tetramethylbenzidine, BD Biosciences). Plates were incubated in the dark at room temperature for approximately 30 minutes, and the reaction was stopped with the addition of 2M H_2_SO_4_ (Merck). Absorbance was measured at 450nm using a microplate reader (BMG LABTECH).

**Focus Forming Inhibition Assay (FFIA)**

Vero cells were plated in 96-well flat-bottom plates at 5 x 10^4^ cells/well and allowed to adhere overnight using similar conditions as described above. Live and γ-NDV were activated with 10 μg/mL of Tosyl phenylalanyl chloromethyl ketone (TPCK)-trypsin for 30 minutes and then diluted in FFA media (DMEM + HEPES (Gibco) + 1% P/S + 1% L-Glu). Immune sera were incubated at 56°C for 30 minutes to inactivate complement and then serially diluted in PBS prior to mixing with diluted virus in a 1:1 ratio to give a final multiplicity of infection (MOI) of 0.1. Mixture of virus:sera was added to confluent monolayers (in triplicate). PBS combined with allantoic fluid diluted in FFA media was also added to control wells. Monolayers were incubated for 2 hours at 37°C, the supernatant was discarded, cell monolayers were washed, and fresh FFA media was added. Plates were incubated for a further 22 hours at 37°C in 5% CO_2_. For visualisation, cells were fixed and permeabilised with cold acetone:methanol (1:1) for 15 minutes at 4°C. Monolayers were subsequently washed with PBS, and treated with mouse anti-NDV serum (1:200 dilution in PBS + 1% BSA) for 1 hour at 4°C, followed by staining with Alexa-Fluor® 488-conjugated anti-mouse IgG (H+L) secondary antibody (to be visualised via the FITC-channel) for 1 hour at 4°C in the dark. Cell nuclei were then stained with 1 μg/mL of DAPI (ThermoFisher). Plates were visualised using Nikon TiE inverted fluorescence microscope and analysed using NIS elements software (Tokyo, Japan).
